# Effects of topical mechanical stability on the formation of Masquelet membrane in a rabbit radial defect model

**DOI:** 10.1038/s41598-020-76112-3

**Published:** 2020-11-03

**Authors:** Jie Xie, Donghao Liu, Haoyi Wang, Haitao Long, Yong Zhu, Yihe Hu, Min Zeng

**Affiliations:** Department of Orthopedics, Xiangya Hospital, Central South University, No. 87 Xiangya Road, Changsha, 410008 Hunan China

**Keywords:** Trauma, Experimental models of disease, Preclinical research

## Abstract

The exact mechanism of Masquelet technique is unknown. This study intends to explore the effects of topical mechanical stability on the formation of Masquelet membrane. Segmental radius shaft defect was created in all rabbits, which were filled with polymethylmethacrylate (PMMA) in Non-fixation group, and with PMMA fixed with plates in Fixation group, and subjected to no disposal in control group. The topical stability of PMMA and plates were monitored via X-ray and mechanical test. And the membranes were excised for further Histological, IHC and Western-Blotting analysis 4 and 6 weeks post-operatively*.* X-ray revealed no sign of plates loosening, or shift of PMMA. Mechanical tests revealed superior topical stability by plates. Pathological examinations suggested that vascularized and osteogenic membranes were formed around PMMA. IHC and Western-Blotting analysis revealed that both Fixation and Non-fixation group exerted significant effects on the expression of Ki67, COL I, and CD31 positive cells, as well as the protein expression of osteogenic (RUNX2, ALP) and angiogenic (VEGFA, TGF-β1) factors. And compared with membrane in Non-fixation group, Fixing PMMA spacer with plates caused a significant increase in osteogenic and angiogenic expression. This study indicates that rigid fixation provided by plate in Masquelet technique positively alters the quality of membrane formed surrounding PMMA, in terms of significantly osteogenic and angiogenic potential.

## Introduction

Segmental bone defects, caused by trauma, osteomyelitis, and tumor resection, has become a major health problem obfuscating orthopedist^1–3^. One strategy of growing concern in bone defect repairing is the Masquelet or induced membrane technique^4–6^. Mixed results are shown in previous animal or human studies^1,3,7,8^. The key to bone repair relies on a vascularized and osteoblastic membrane formed during the first stage of Masquelet technique^9,10^. Still, the exact generating and regulatory mechanism of Masquelet membrane are unknown. In order to solve these problems, several studies show that the membrane has structural characteristics and osteogenic potentials. It is not just offering a physical barrier and a rich vascular network, but also numerous osteoprogenitor cells and a local source of key biochemical factors, such as BMP2, and TGF-β^11–13^. And previous researches have demonstrated that selecting a new model, such as spacer alternatives, micro-topography modification, local antibiotic usage, would affect the structure and function of membrane^13–18^.

During the first phase of Masquelet, the bone defects filling with bone cement (polymethyl methacrylate, PMMA) spacer are fixed with internal plate, intramedullary nail, external fixator, casting, or nothing^12,17,19–21^. But it is not sure that which fixation method is more appropriate for membrane formation in the first stage of Masquelet^13,22–24^. It is known that the stiffness of fixation construct directly affects the fretting of fracture fragments, subsequently impacts the healing of bone fracture. A relatively high level of rigid fixation results in intramembranous ossification, while less rigid fixation results in cartilaginous callus and endochonrdral ossification^25,26^. And there is no literature reported regarding whether there are differences in the membrane formation among various fixation pattern.

This study intends to explore the effects of topical mechanical stability on the formation of Masquelet membrane. Based on previous studies, we hypothesize that an increase in fixation strength will results in superior Masquelet membrane with significance in osteogenic and angiogenic factors expression.

## Materials and methods

### Animal models

All procedures were done with the approval of the Medical Ethics Committee of Central South University, and all experiments were performed in accordance with the guidelines and regulations of our Medical Ethics Committee. Thirty-six New Zealand white rabbits, weighing approximately 2500–3000 g and aged 24–28 weeks, were evenly randomized into three groups (Fixation group, Non-fixation group, and control group). Segmental left radius shaft defect (length, 9 mm) was created in all rabbits. And the bone defects were filled with PMMA (length, 9 mm) only (Non-fixation group, n = 12), or PMMA fixed with plates (Synthes, USA) (Fixation group, n = 12), respectively. In the control group, the defects were subjected to no disposal (control group, n = 12).

**Figure 1:**
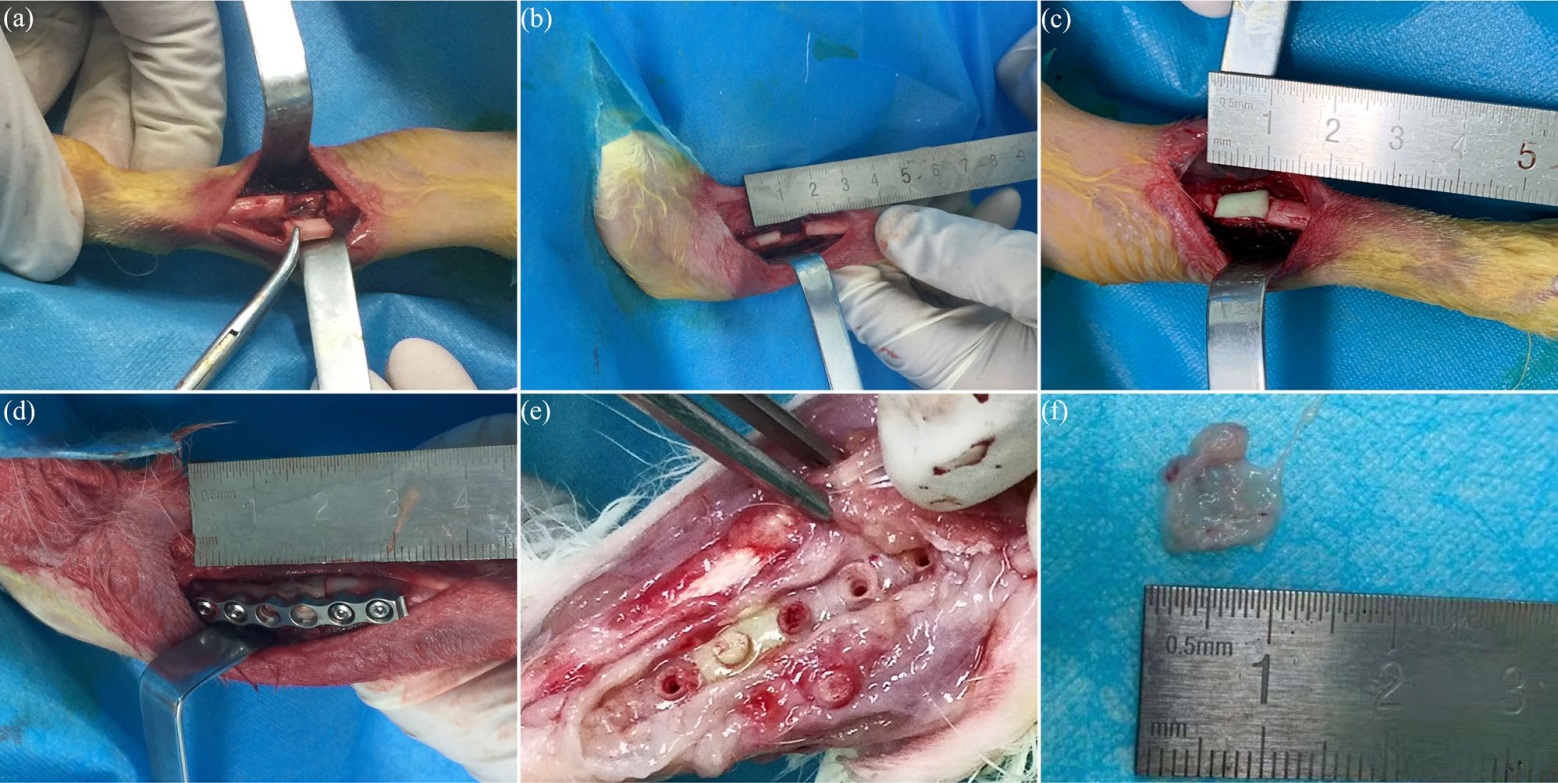
Application of the Masquelet technique. (**a**,**b**) Segmental left radius shaft defect (length, 9 mm) was created. (**c**) Bone defect was filled with PMMA spacer (3 mmØ × 9 mm). (**d**) PMMA was fixed with stainless steel plates. (**e**,**f**) Masquelet membrane was formed surrounding the cement, 4 or 6 weeks postoperatively.

Xylazine hydrochloride was given to rabbits by intramuscular injection, and Iidocaine was used for additional regional anesthesia before osteotomy. All rabbits were arranged on fixed frames, and the left forelimbs were shaved, prepared, and draped before surgery. After the skin, superficial fascia and muscles of the left forelimb were incised, exposing the shaft of radius. A 9 mm defect was created at approximately middle radius shaft with a high-speed power drill, and subsequently a cylindrical PMMA spacer (3 mmØ × 9 mm) was placed in the defect in Fixation and Non-fixation group. A six-hole, 2.5 mm stainless steel plates (Synthes, USA) was applied to the radius shaft and secured in place with two proximal and two distal 1.5 mm cortical screws in Fixation group. Then tight suture of the fascia incision with 4–0 absorbable sutures ensures stability of PMMA. The skin wounds were closed with interrupted 4–0 Mersilk sutures. Cefotaxime Sodium 20,000 IU/kg was administered intramuscularly immediately, 24 and 48 h post-operatively (Fig. 1a–d).

All animals survived till the end of the research. Immediately, 4 and 6 weeks post-operatively, X-ray was used to evaluate the location of implants. Half of animals were sacrificed at 4 weeks after operation, and the other half were sacrificed at 6 weeks through air embolism. Through the former incision, the skin and muscles were dissected. Membranes formed around PMMA were excised completely. The membranes were divided into two parts, which were respectively fixed in 10% paraformaldehyde for later pathological examination and shock frozen (− 80 °C) for later Western-Blotting analysis (Fig. 1e,f).

### Topical mechanical stability

The stability of PMMA and plates (i.e. PMMA migration and plates loosening) were monitored via X-ray in all rabbits immediately, 4 and 6 weeks post-operatively.

**Figure 2:**
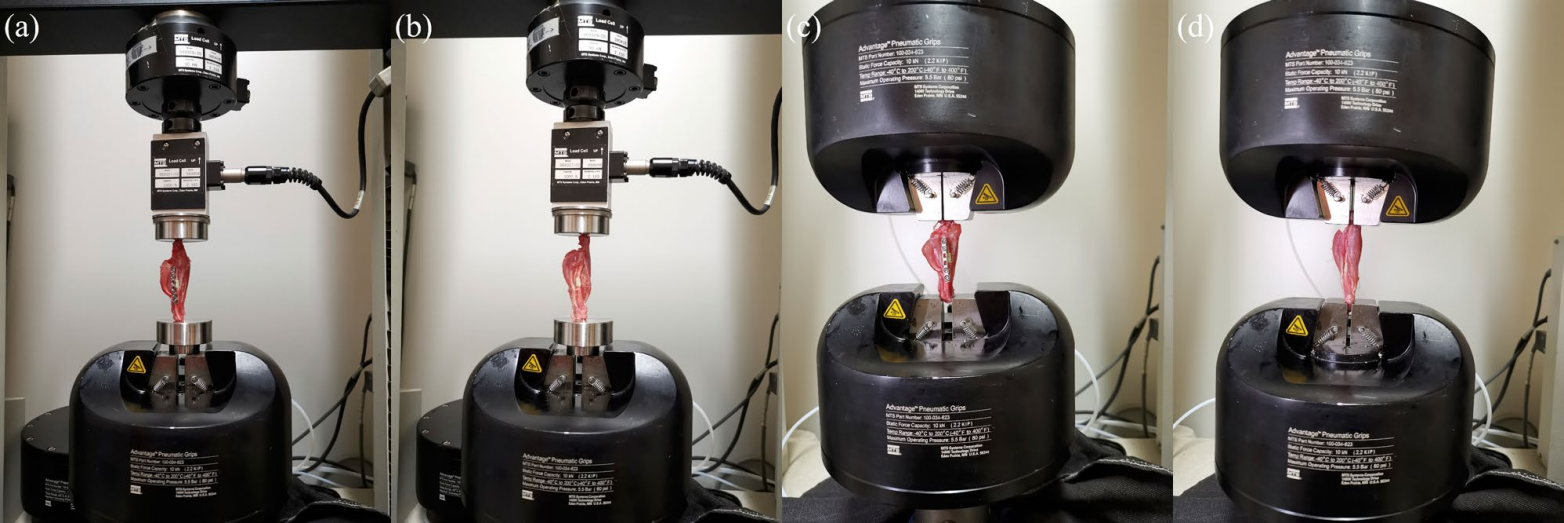
Mechanical tests. (**a**) Compression test in Fixation group. (**b**) Compression test in non-fixation group. (**c**) Tensile test in Fixation group. (**d**) Tensile test in non-fixation group.

And another twelve integral forearms, with a 9 mm defect at middle radius shaft, were used for topical mechanical stability. As mentioned above, half defects were filled with PMMA only, and the other half were filled with PMMA fixed with plates. The compression and tensile tests were performed with a mechanical test machine (MTS Systems Corporation, USA), with a velocity of 2 N/mm (Fig. 2). The modulus and peak load data were collected from the characteristic load–displacement curves. Modulus was defined as the slope of the curve, and peak load as a discontinuity in the monotonic curve, usually denoted by a sharp decrease in the force.

### Histological and IHC analysis

After embedding and sectioning, paraffin specimens were heated and subsequently removed by sequential washes (xylene 100%, 20 min thrice; alcohol 100%, 5 min: alcohol 95%, 5 min; alcohol 85%, 5 min; alcohol 75%, 5 min; distilled water, 5 min). Subsequently, the specimens were stained with haematoxylin/eosin (HE) and Alizarin red S to evaluate the histopathological characteristics. And the amount of calcification was examined quantitatively at × 100 magnification (n = 6).

Content of cell proliferation, osteogenesis, and vascularization in the induced membranes were measured using immunohistochemistry (IHC) analysis by detection of Ki67, COL I, and CD31 (n = 6/group), respectively. Paraffin sections were first removed by sequential washes (xylene 100%, 20 min thrice; alcohol 100%, 5 min: alcohol 95%, 5 min; alcohol 85%, 5 min; alcohol 75%, 5 min; distilled water, 5 min). Then, the processes of antigen retrieval, incubation of primary antibody (CD31, Ki67, and COL I), incubation of secondary antibody (anti-mouse, rabbit, mouse-IgG antibody-HRP polymer) were in progress. Finally, after DAB coloration, hematoxylin redyeing, and Alcohol dehydration, IHC sections were imaged under microscope (DMIL-LED, LEICA, Germany). An independent observer blinded to the group constituency analysed the images. The IHC results were examined quantitatively at × 400 magnification for cell proliferation, osteogenesis, and vascularization existence, and the positive immunostaining cell counts were performed in 6 independent regions.

### Western blot analysis

Osteogenic proteins (RUNX2 and ALP) and angiogenic proteins (VEGF and TGF-β1) were measured using Western blot analysis in 4 and 6 weeks postoperatively. Western blot was performed according to the manufacturer’s protocol. The primary antibodies including anti-RUNX2 (1:1000), anti-ALP (1 μg/ml), anti-VEGF (5 μg/ml), anti-TGF-β1 (1:300), and β-actin (1:5000) were obtained from Abcam (Cambridge, UK). The immunoreactive bands were visualized and finally quantified using Quantity One Software. And all values were normalized to the value of β-actin.

### Statistical analysis

All data were reported as means with standard deviations, and analyzed by SPSS 13.0 software (SPSS Inc, Chicago, IL, USA). Differences were considered as significant at *P* ≤ 0.05. For normally distributed data, Student t test or one-way analysis of variance was used to compare differences between 2 different groups or among more than 2 groups.

## Results

### Topical mechanical stability

**Figure 3:**
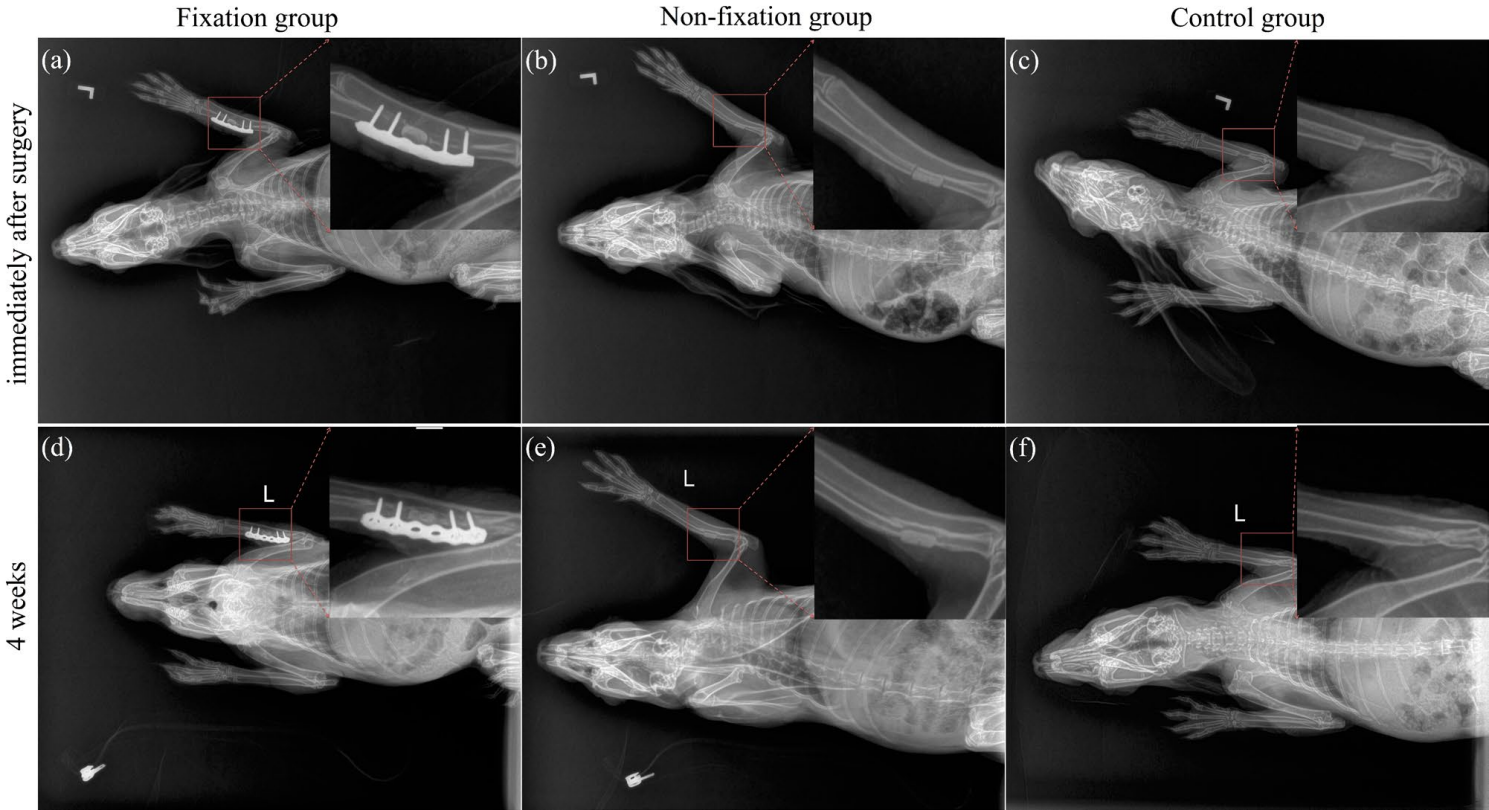
Postoperative X-ray images. (**a**–**c**) X-ray images shown that segmental left radius shaft defects were respectively fixed with PMMA and plates, PMMA only, or nothing, immediately after surgery. (**d**–**f**) There was no sign of plates loosening, or shift of PMMA, or immigration of fracture ends, 4 weeks postoperatively.

Immediately after surgery, as presented in Fig. 3a–c, segmental left radius shaft defects were fixed with PMMA and plates, PMMA only, or nothing, suggesting that these models were successful. Both plates and PMMA were radiopaque so that the bone defects and the implant materials were clearly visible. Compared with immediate and 4 weeks postoperative radiographs (Fig. 3a–f), there was no sign of PMMA displacement. And the reduction of both ends of fracture was in accordance with that immediately after operation indicated that there was no significant movement between fracture ends.

**Figure 4:**
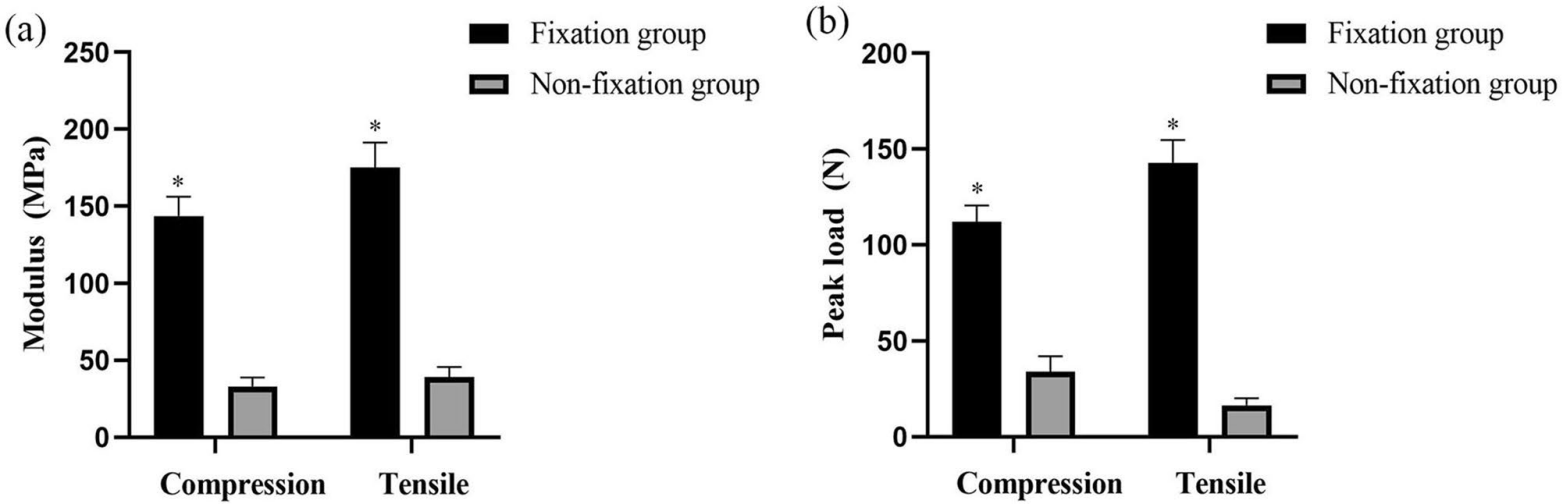
The quantitative analysis of mechanical tests (Compression and Tensile Tests). Significant differences between the Fixation group and Non-fixation group are indicated as * (*P* < 0.05). For each group, n = 6.

The quantitative analysis of mechanical test showed that the compression and tensile modulus in Fixation group were significantly higher than that of the Non-fixation group, and that the compression and tensile peak load in Fixation group were significantly higher than that of the Non-fixation group (Fig. 4), which indicated that the internal plate could provide additional topical stability.

### Gross histology

**Figure 5:**
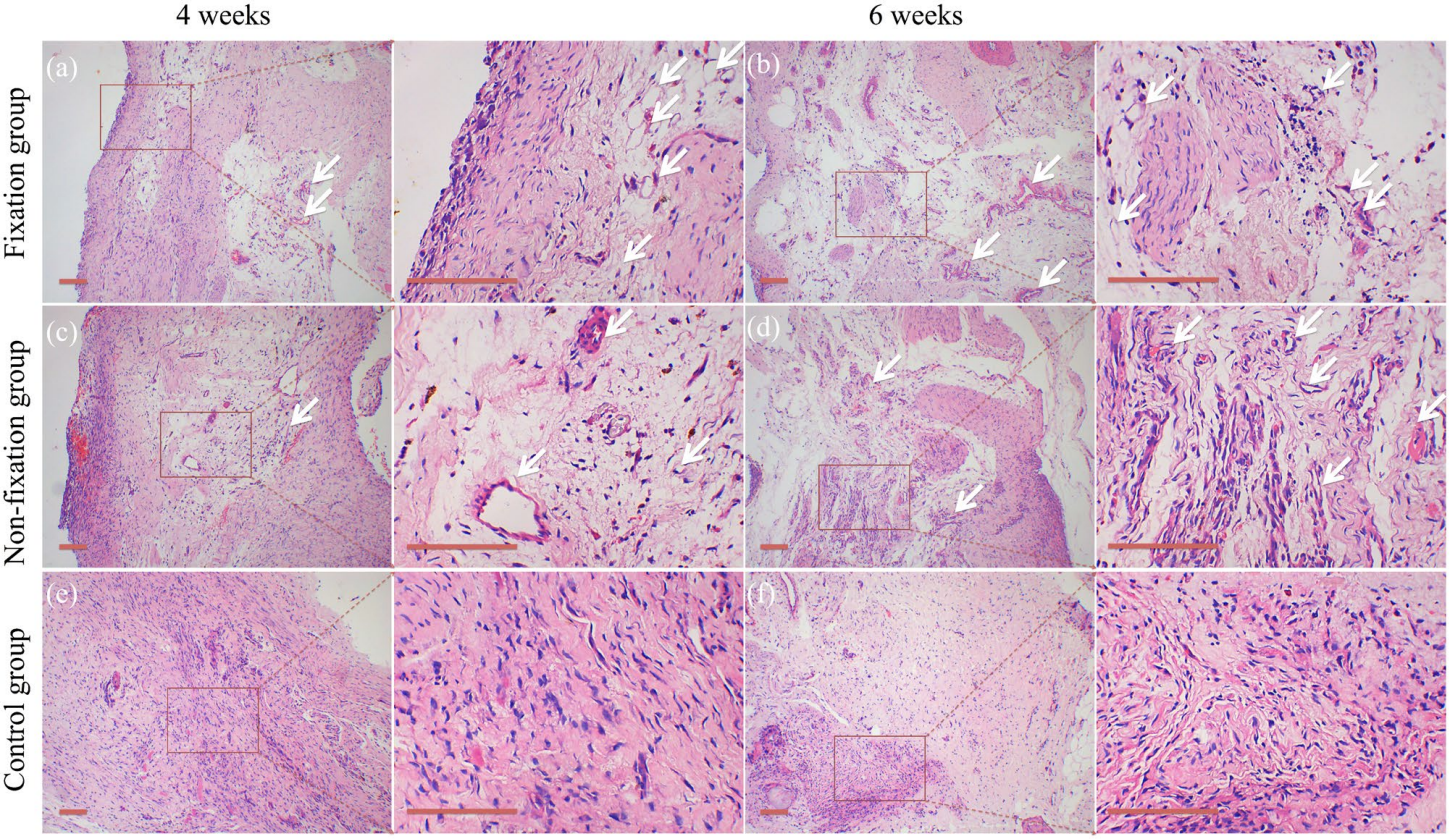
Representative H&E staining of Masquelet membrane (scale bar: 100 mm). H&E staining shown that the intensive fibrous, cell-rich, and vascularized tissue (white arrow) were formed in Fixation group (**a**) and Non-fixation group (**c**), while just little vessel like structure was observed in control group (**e**), in 4 weeks postoperatively. The micro-vessels had a tendency to increase and to be more mature in Fixation group (**b**) and Non-fixation group (**d**), while still little in control group (**f**), in 6 weeks postoperatively.

**Figure 6:**
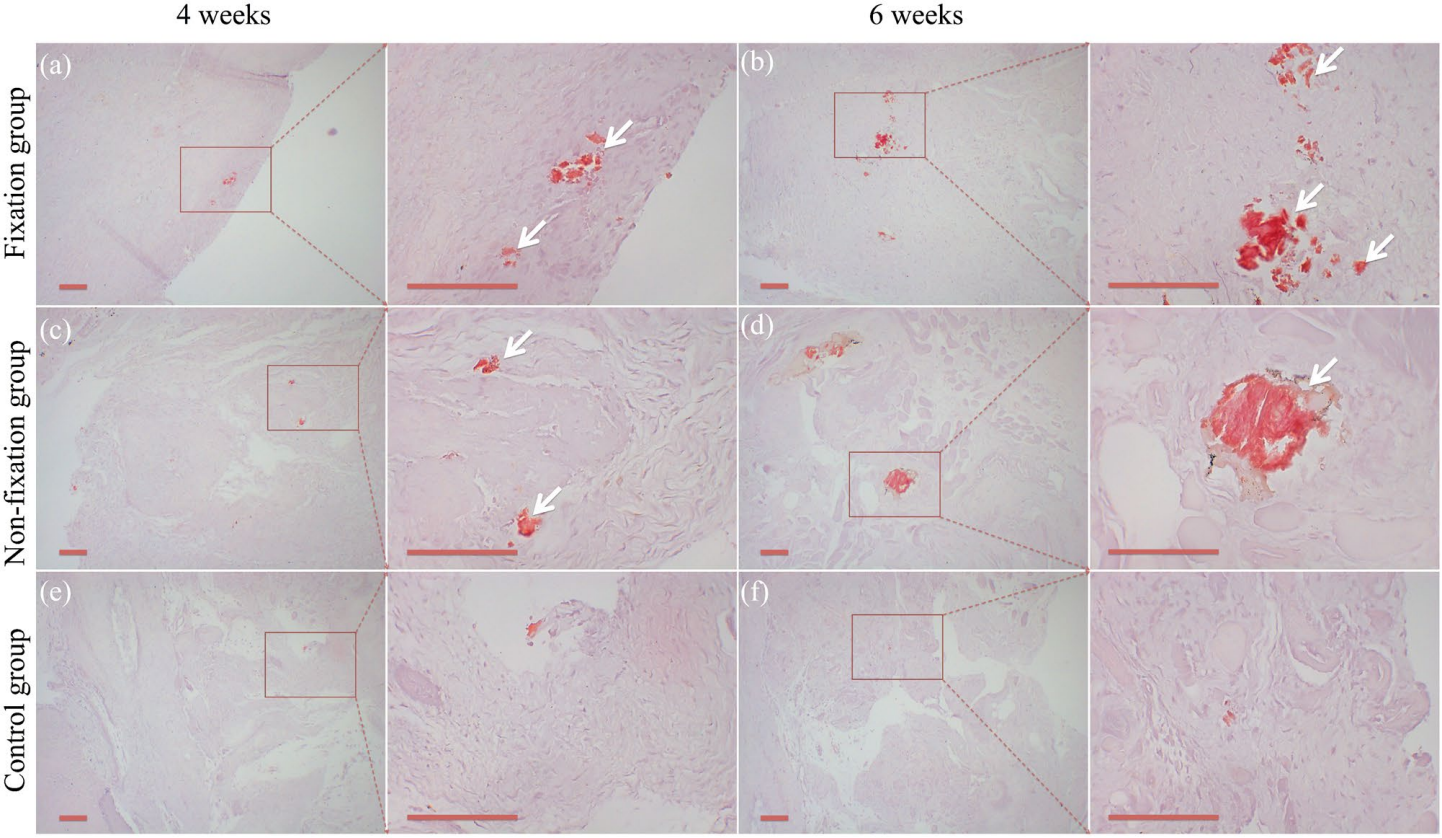
Representative Alizarin red staining of Masquelet membrane (scale bar: 100 mm). Calcium deposition (white arrow) was observed in Fixation group (**a**,**b**) and Non-fixation group (**c**,**d**), while just little in control group (**e**,**f**), 4 and 6 weeks post-operation, respectively.

**Figure 7:**
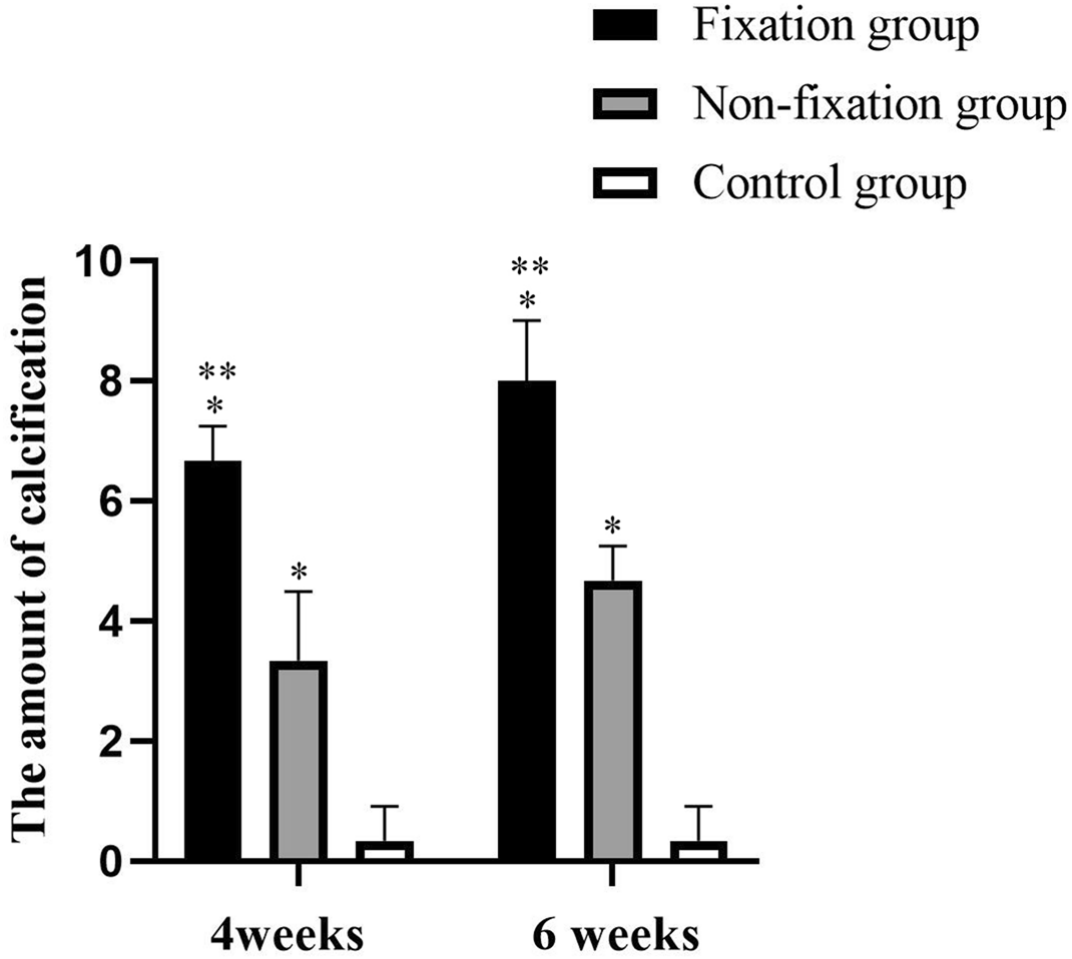
The quantitative analysis of calcification in Alizarin red staining. Significant differences between PMMA groups (Fixation group and Non-fixation group) and the control group groups are indicated as * (*P* < 0.05). Significant differences between Fixation group and Non-fixation group are indicated as ** (*P* < 0.05). For each group, n = 6.

Gross observation showed the translucent and elastic membrane was formed around PMMA (Fig. 1f). In the control group, there was no membrane formation but callus tissue surrounding with connective tissue. The induced membrane (Fixation group and Non-fixation group) and connective tissue (control group) was removed completely for further testing. As shown in Fig. 5, H&E staining revealed an intensive fibrous, cell-rich, and vascularized tissue in PMMA groups (Fixation group and Non-fixation group), while just little vessel like structure was observed in control group. Compared with membrane in Non-fixation group, there were more micro-vessels in Fixation group. And with the extending of implantation time, the micro-vessels had a tendency to increase and to be more mature. The osteogenesis activity was assessed via Alizarin red staining for calcium deposition (Fig. 6). The quantitative analysis of calcification showed that the reaction of calcifying nodules in Fixation group and Non-fixation group was significantly higher than that of the control group, indicating significant osteogenic activity around PMMA. And the amount of calcification in Fixation group was significantly more than that in Non-fixation group in 4 weeks and 6 weeks after operation (Fig. 7).

### Proliferation

**Figure 8:**
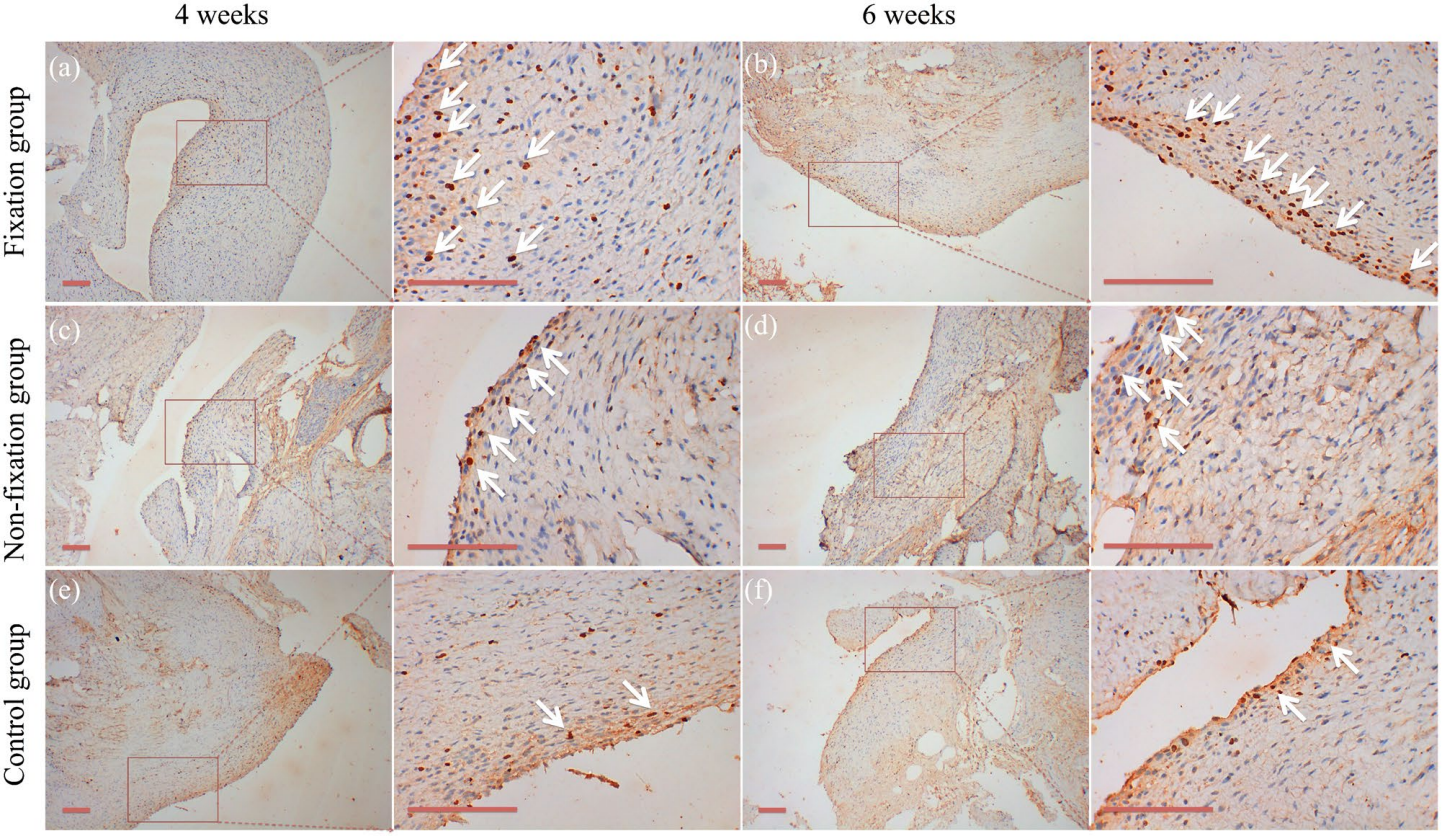
Representative IHC analysis of Ki67 (scale bar: 100 mm). (**a**–**f**) The Ki67 positive cells displayed brown yellow or brown particles (white arrow), and mainly distributed in the periphery of membrane in 4 or 6 weeks postoperatively.

**Figure 9:**
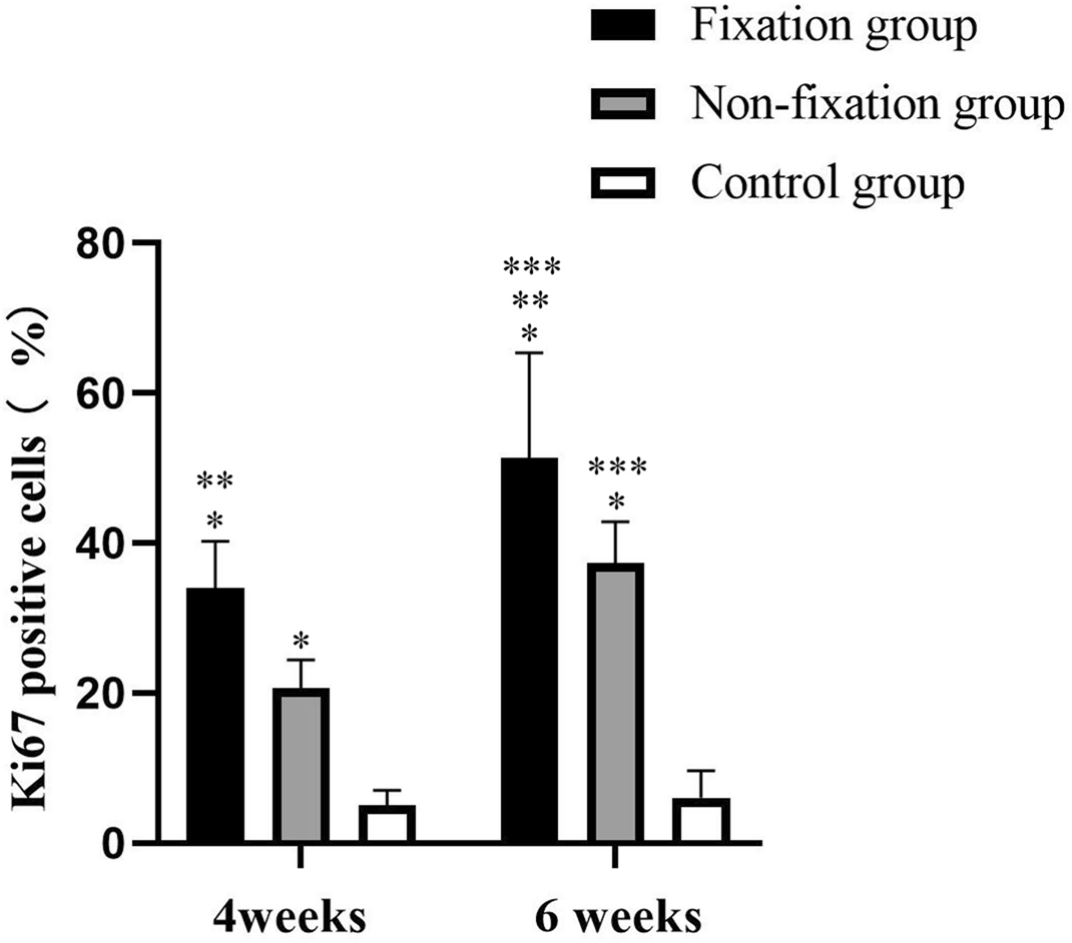
The quantitative analysis of Ki67 positive cells. Significant differences between PMMA groups and the control group groups are indicated as * (*P* < 0.05). Significant differences between Fixation group and Non-fixation group are indicated as ** (*P* < 0.05). Significant differences between 4 and 6 weeks postoperatively in the same group are indicated as *** (*P* < 0.05). For each group, n = 6.

Content of cell proliferation in the induced membranes and in the connective tissue were measured using IHC analysis by detection the percentage of Ki67 positive cells. As shown in Fig. 8, the positive cells displayed brown yellow or brown particles, and mainly distributed in the periphery of membrane. The quantitative results indicated that Ki67 positive cells were significantly higher in the induced membranes than that in the connective tissue. And a significantly increased proliferative activity was observed in membranes of Fixation group in comparison to that of Non-fixation group. A qualitative trend toward increased percentage of Ki67 positive cells was observed in PMMA groups between 4 and 6 weeks (Fig. 9).

### Osteogenesis

**Figure 10:**
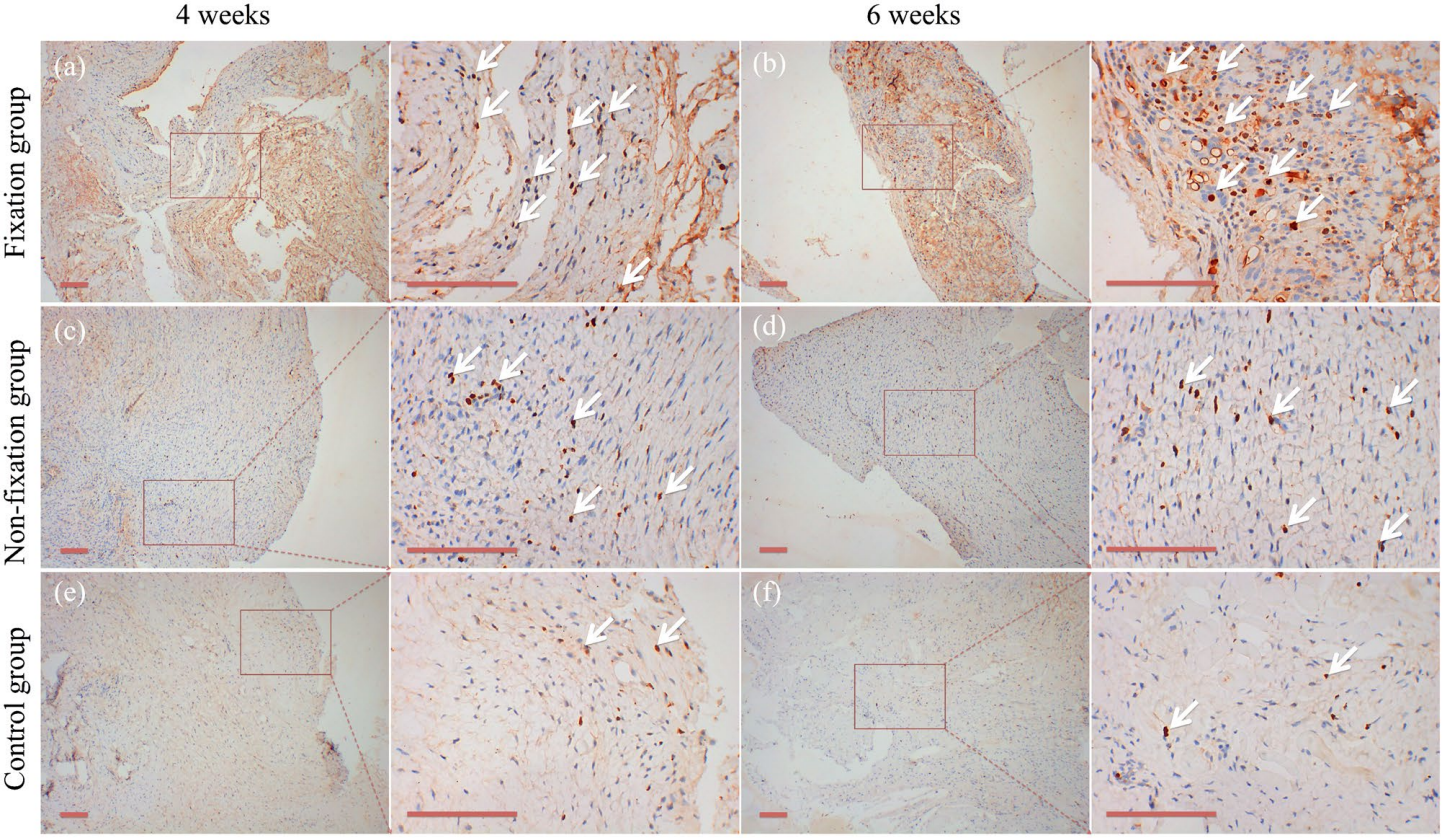
Representative IHC analysis of COL I (scale bar: 100 mm). (**a**–**f**) The COL I positive cells displayed brown yellow or brown particles (white arrow), and mainly scattered in the membrane in 4 or 6 weeks postoperatively.

**Figure 11:**
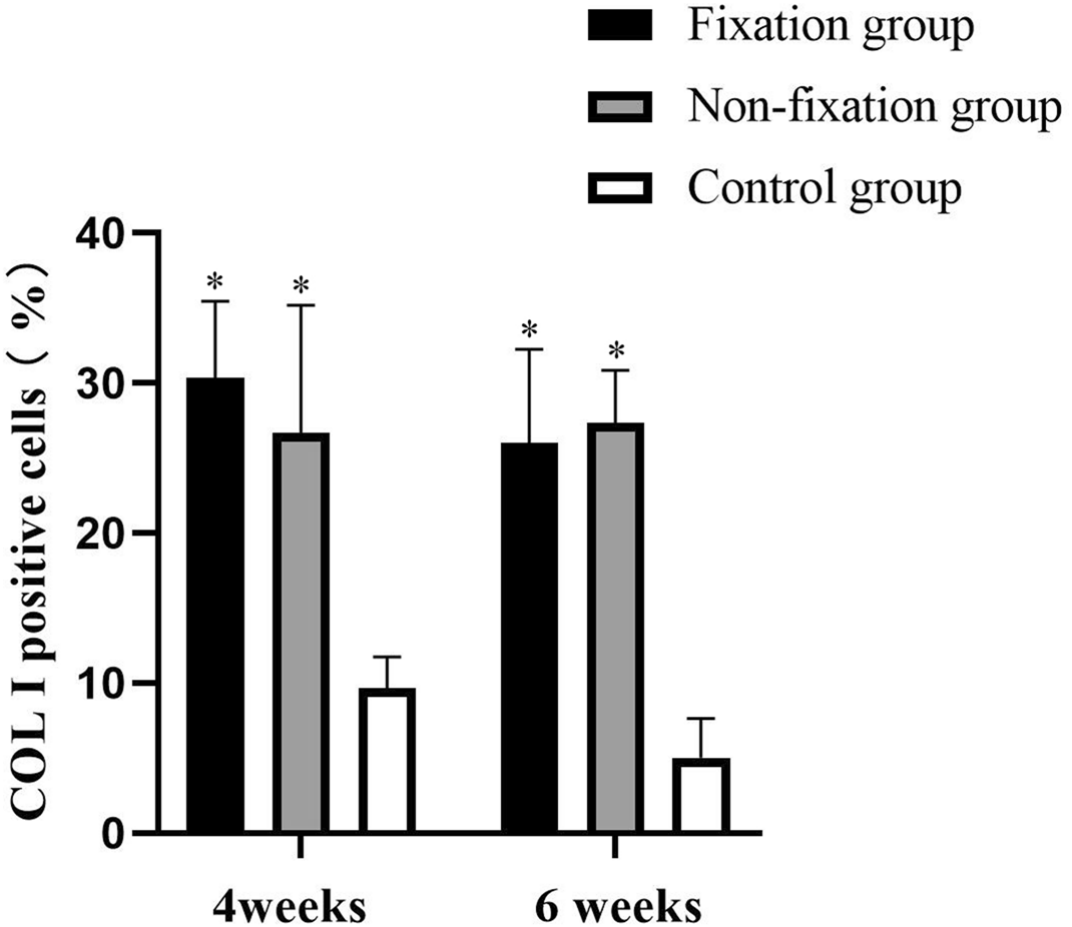
The quantitative analysis of COL I positive cells. Significant differences between PMMA groups and the control group groups are indicated as * (*P* < 0.05). Significant differences between Fixation group and Non-fixation group are indicated as ** (*P* < 0.05). Significant differences between 4 and 6 weeks postoperatively in the same group are indicated as *** (*P* < 0.05). For each group, n = 6.

Content of osteogenesis in the induced membranes and in the connective tissue were measured using IHC analysis by detection COL I. As shown in Fig. 10, the positive cells displayed brown yellow or brown particles, and mainly scattered in the membrane. The qualitative IHC analysis shown that the expression of COL I increased evidently than that of the control group, and that there was no obvious difference in the percentage of COL I between the two PMMA groups in 4 weeks and 6 weeks after operation (Fig. 11).

**Figure 12:**
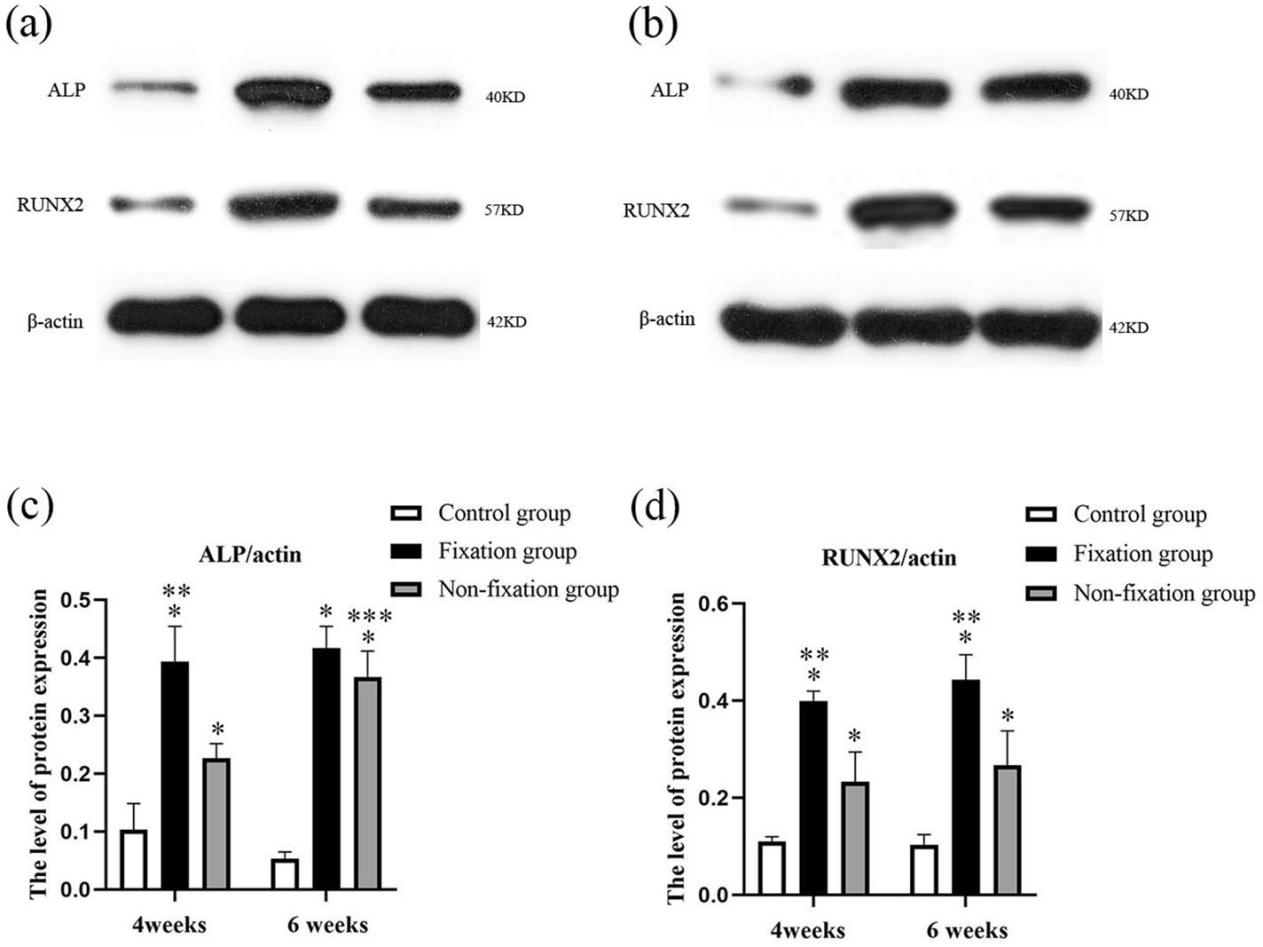
Western-Blotting analysis of osteogenic proteins (RUNX2 and ALP). These blots of each marker were original and not previously cropped. (**a**) 4 weeks postoperatively. (**b**) 6 weeks postoperatively. (**c**,**d**) the quantitative analysis of RUNX2 and ALP. Significant differences between PMMA groups and the control group groups are indicated as * (*P* < 0.05). Significant differences between Fixation group and Non-fixation group are indicated as ** (*P* < 0.05). Significant differences between 4 and 6 weeks postoperatively in the same group are indicated as *** (*P* < 0.05). For each group, n = 6.

As shown in Fig. 12, Western blot analysis revealed that the membrane extracted from Fixation group and Non-fixation group both exhibited higher protein expression of ALP and Runx2 compared to the control group, 4 and 6 weeks postoperatively. Additionally, in comparison to membrane in Non-fixation group, a significant increasing trend in the protein expression of ALP and Runx2 was observed in Fixation group, although the difference of ALP expression did not reach significance in 6 weeks postoperatively. Western blot analysis also revealed that the expression of ALP in 6 weeks postoperatively was significant higher than that in 4 weeks postoperatively in Non-Fixation group.

### Vascularization

**Figure 13:**
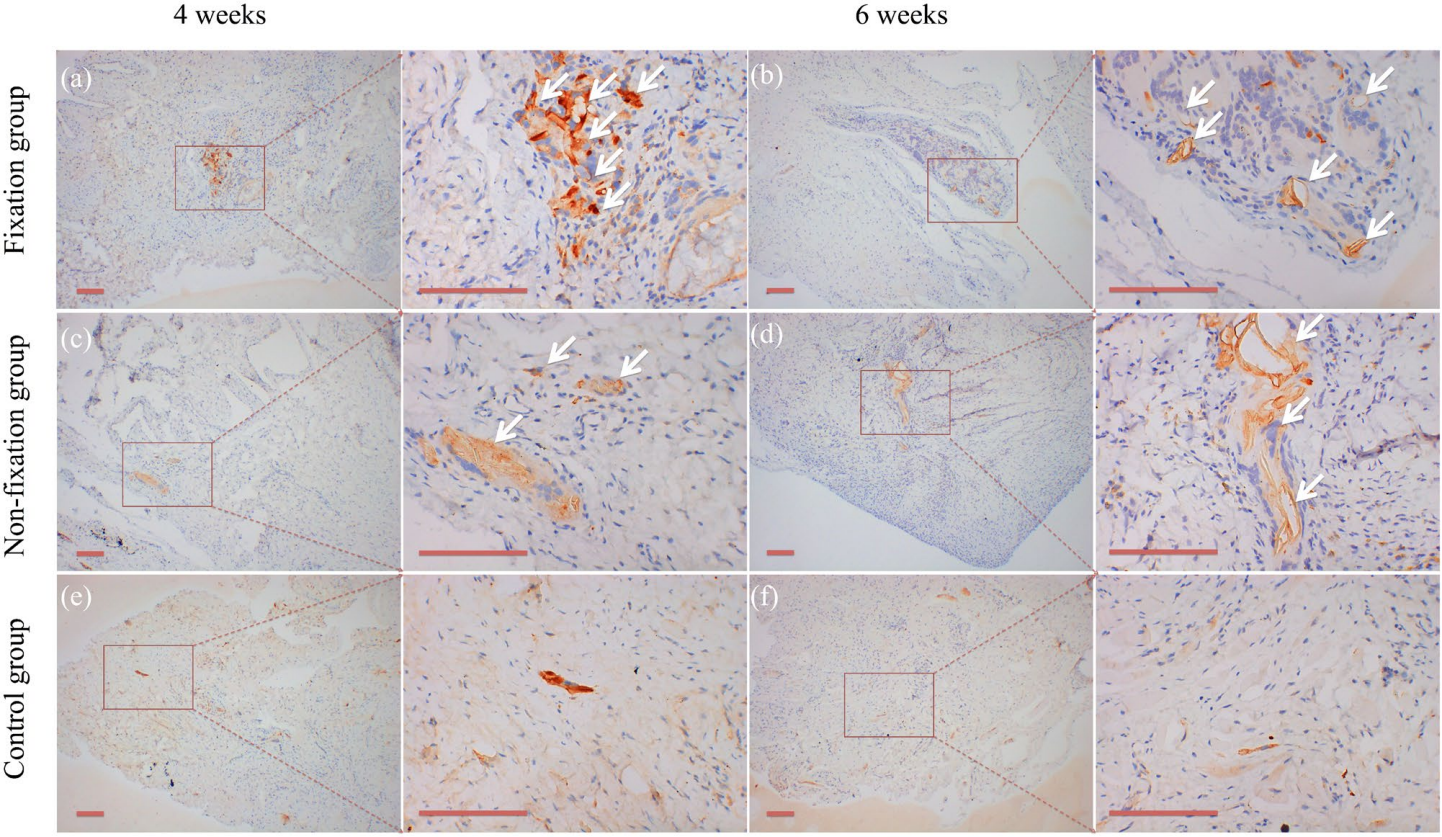
Representative IHC analysis of CD31 (scale bar: 100 mm). (**a**–**f**) The COL I positive cells displayed brown yellow particles (white arrow), mainly scattered, and formed tube-like vascular structure in the membrane in 4 or 6 weeks postoperatively.

**Figure 14:**
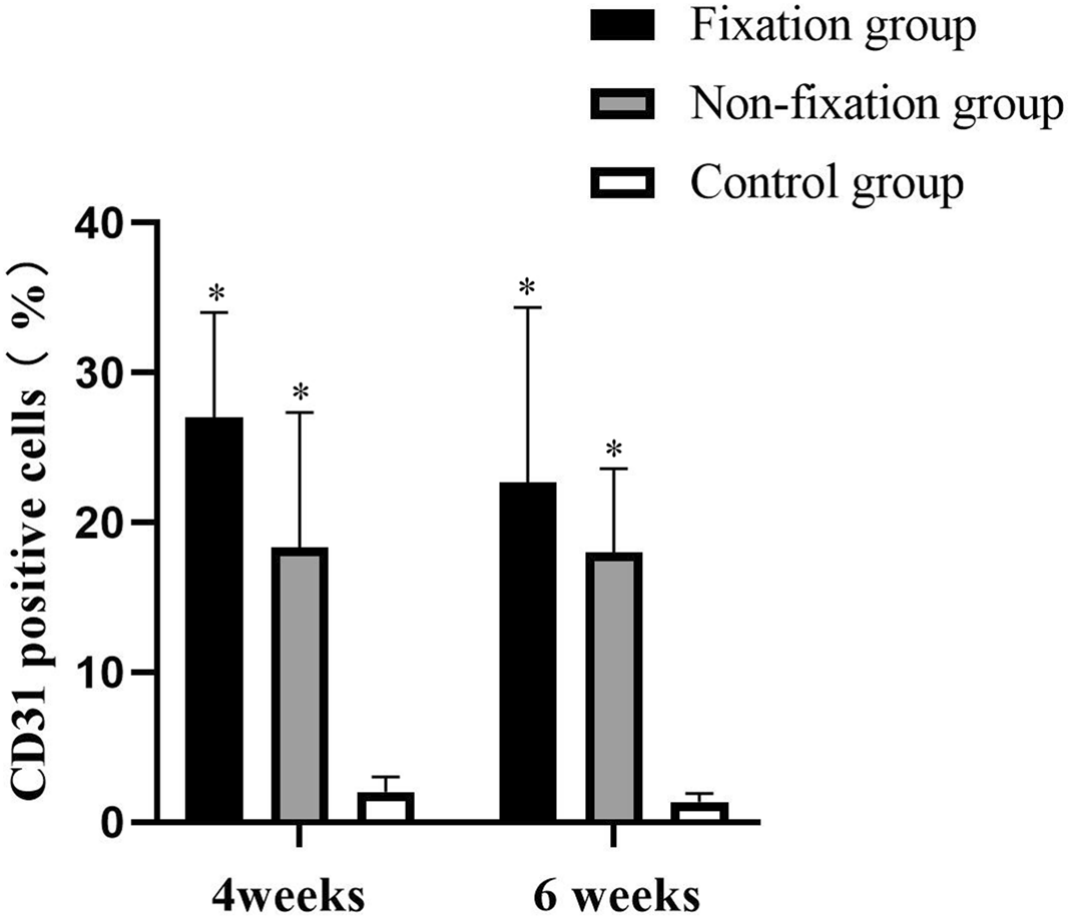
The quantitative analysis of CD31 positive cells. Significant differences between PMMA groups and the control group groups are indicated as * (*P* < 0.05). Significant differences between Fixation group and Non-fixation group are indicated as ** (*P* < 0.05). Significant differences between 4 and 6 weeks postoperatively in the same group are indicated as *** (*P* < 0.05). For each group, n = 6.

Content of vascularization in the induced membranes and in the connective tissue were measured using IHC analysis by detection the percentage of CD31 positive cells. As shown in Fig. 13, the positive cells displayed brown yellow particles, mainly scattered, and formed tube-like vascular structure in the membrane. And with the extending of implantation time, the CD31 positive cells formed the more mature organized micro-vessels in 6 weeks after operation. The qualitative results indicated that CD31 positive cells were significantly higher in the induced membranes, but that in the connective tissue was little. The comparison between Fixation group and Non-fixation group did not reveal any significant differences postoperatively (Fig. 14).

**Figure 15:**
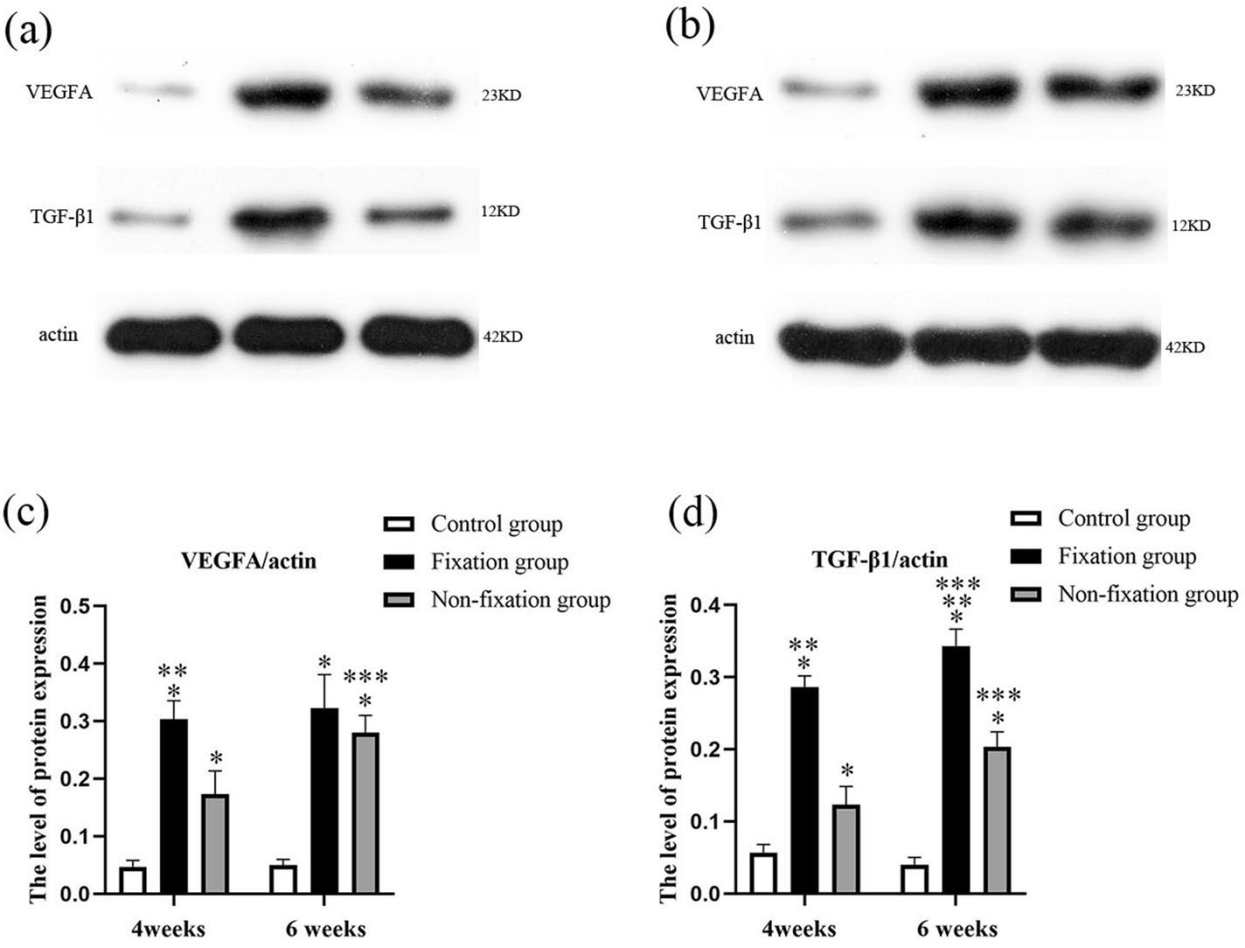
Western-Blotting analysis of angiogenic proteins (VEGFA and TGF-β1). These blots of each marker were original and not previously cropped. (**a**) 4 weeks postoperatively. (**b**) 6 weeks postoperatively. (**c**,**d**) the quantitative analysis of VEGFA and TGF-β1. Significant differences between PMMA groups and the control group groups are indicated as * (*P* < 0.05). Significant differences between Fixation group and Non-fixation group are indicated as ** (*P* < 0.05). Significant differences between 4 and 6 weeks postoperatively in the same group are indicated as *** (*P* < 0.05). For each group, n = 6.

Western-Blotting analysis revealed that both Fixation group and Non-fixation group exert a significant effect on the protein expression of VEGFA and TGF-β1 (Fig. 15). And in comparison to membrane in Non-fixation group, an increasing trend in the protein expression of VEGFA and TGF-β1 was observed in Fixation group, although the difference of VEGFA did not reach significance on 6 weeks postoperatively. Western blot analysis also revealed that the expression of VEGFA in 6 weeks postoperatively was significant higher than that in 4 weeks postoperatively in Non-Fixation group, and that the expression of TGF-β1 in 6 weeks postoperatively was significant higher than that in 4 weeks postoperatively in both Fixation group and Non-fixation group.

## Discussion

There were many researches regarding that the stability of fracture broken end directly affects the healing of bone fracture^25–28^. While during the first phase of Masquelet, the bone defects filling with PMMA spacer are fixed with internal plate, intramedullary nail, external fixator, casting, or nothing^17,19–21^. Each fixed mode could result in a different mechanical environment, subsequently have significant influence on bone repair. No study has been done yet to evaluate the effect of PMMA spacer stability on subsequently bone formation in the membrane cavity. This was the first study to investigate the influencing of topical mechanical stability of fracture broken end on the subsequently formation of Masquelet membrane. In order to explore the specific mechanisms, two fixation variables, rigid fixation offered by plates and relative fixation by suturing, were selected for further research.

The rabbit models of bone defects were commonly used in laboratory experiments, and stainless-steel internal plate was usually applied for the fixation of bone defect ends^9,29,30^. Through X-ray measurement and mechanical tests postoperatively we confirmed that the Masquelet models with plate internal fixation was successfully made. The X-ray results suggested no significant movement between the fracture ends, even in the control group without PMMA and plate. And the mechanical tests revealed that superior topical mechanical stability were achieved by internal plate. Due to the integrity of ulna and interosseous membrane, there was no visible displacement of fracture segments^19^. And due to the supporting of PMMA in defects, and suture tension of fascia, PMMA in Non-fixation group was in relatively stable pattern with little distortions^30^. Still, even in Fixation group, there could have been micro-motions of fracture fragments causing fretting, which might have an effect on the subsequently membrane formation.

Through gross histology we found that a translucent and membrane-like fibrotic capsule was formed around PMMA, and that connective tissue formed in the bone defects in the control group, which is consistent with the literature reported^15,19^. And, the quantitative analysis of calcification indicated significant osteogenic activity around PMMA. H&E and Alizarin red staining results suggested that the intensive fibrous, cell-rich, calcium-positive, and vascularized membrane around PMMA would be more conducive to osteogenic activity.

Content of proliferation was assessed by detection the percentage of Ki67 positive cells. Ki67 is a core protein seen in proliferating cells, which exhibits good morphological properties of cell proliferation^31^. The proliferation was significantly higher in the induced membranes compared to the control groups, and a significantly increased proliferative activity was observed in membranes of Fixation group in comparison to that of Non-fixation group, suggesting that the mechanical stability offered by plates was beneficial to cell proliferation in the induced membrane. The results was corresponding to previous research that the cell proliferation was relatively active in the membrane^15,19^. Besides, our research found that a quantitative trend toward increased proliferation was observed in PMMA groups between 4 and 6 weeks.

The Col I was selected for osteogenesis qualitatively. And the expression levels of ALP and RUNX2 were used for the quantitative analysis of osteogenic activity, of which ALP is an early sign of osteoblast differentiation and maturation, and RUNX2 plays a central role in coordinating multiple signals involved in osteoblast activity^32^. By comparing results between PMMA groups and the control group, osteogenic activity of the induced membranes was obviously expressed during the whole observation period. Similar animal results were obtained from previous studies. It is well-known that the different mechanical stabilities around bone defects lead to different callus responses and osteoblast activities^33^. And current clinical findings indicate that appropriate fixation of the bone defect is desirable in Masquelet technique, with the use of internal fixator, external fixator, or plaster cast^1,3,7^. Still, the exact mechanism remains unknown. In our research, an increasing trend in the protein expression of ALP and Runx2 was observed in Fixation group, in comparison to Non-fixation group. These results revealed that the rigid fixation provided by internal plate could further promote osteogenesis, which implying that the use of fixation in Masquelet technique has potential clinical benefits.

The detection of CD31 positive cells represents the formation of new vessels. And the vascularity of the membrane was quantitatively evaluated by detecting the level of VEGF and TGF-β1^16,34^. Previous researches have reported that the tube-like vascular structure is formed in the induced membrane around PMMA, and the highly vascular membrane has many similarities to the periosteum^13,35^. The present study indicated that CD31 positive cells were relatively high in the induced membranes, and the higher level of CD31 expression signals greater capacity of vascularization. And the relative expression levels of VEGF and TGF-β1 in membrane around PMMA were higher than those in connective tissue of the control group. Besides, significant differences were found between Fixation and Non-fixation group, suggesting that the rigid fixation provided by internal plate could further promote vascularization too.

Exploring the influencing of topical mechanical stability on subsequently membrane formation could help to further the clinical applications. Further experiments and clinical research to verify their effectiveness would help to further understand the exact generating and regulatory mechanism in Masquelet technique and help to improve clinical outcomes. And the next step in this research would be to further validate if rigid fixation benefits bone defect repairing in the second stage of Masquelet. Still, due to the foreign-body reaction of implant, the internal plate induces its own membrane, increasing the risk of misinterpretation of the data collection^13,35^. And more control groups (e.g. defects fixed with plates only) are needed to rule out the interference in further experiments.

## Conclusion

The results of the current study clearly indicate that rigid fixation provided by internal plate in Masquelet technique positively alters the quality of membrane formed surrounding PMMA, in terms of significantly osteogenic and angiogenic factors expression, and greater potency of bone formation in the second stage of Masquelet. Specific as follow: Membrane surrounding PMMA has some osteogenic and angiogenic potential in Fixation group and Non-fixation group; Compared with membrane in Non-fixation group and Control group, pathological examinations suggest that the relatively more vascularized and osteogenic membrane was formed in Fixation group, and IHC results suggest that the expression of Ki67, COL I, and CD31 positive cells increased evidently; Fixing PMMA spacer with internal plate causes a significant increase in osteogenic factors (RUNX2 and ALP) and angiogenic factors (VEGF and TGF-β1) in 4 and 6 weeks postoperatively.

## Supplementary information

Supplementary Information
