# Supplementary material for: Effects of topical mechanical stability on the formation of Masquelet membrane in a rabbit radial defect model
Source: Sci Rep. 2020 Nov 3;10:18939. doi: 10.1038/s41598-020-76112-3 (PMC7609590; doi:10.1038/s41598-020-76112-3)
Supplement: Supplementary file 1 — Supplementary Information [file 41598_2020_76112_MOESM1_ESM.docx]

**Effects of Topical Mechanical Stability on the Formation of Masquelet Membrane in a Rabbit Radial Defect Model**

Jie Xie^1^, Donghao Liu^1^, Haoyi Wang^1^, Haitao Long^1^, Yong Zhu^1^, Yihe Hu^1^, Min Zeng^1^,

^1^Department of Orthopedics, Xiangya Hospital, Central South University, No. 87 Xiangya Road, Changsha, Hunan 410008, China

Correspondence to: Min Zeng, e-mail: xy_zengmin@163.com; Tel: +86 18874034482 Fax: 0731- 89753005

**Original blots**

**(1) 4 weeks postoperatively**

**ALP
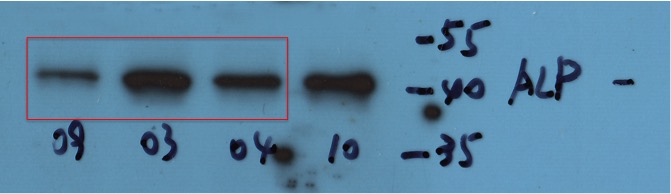
**

**RUNX2**


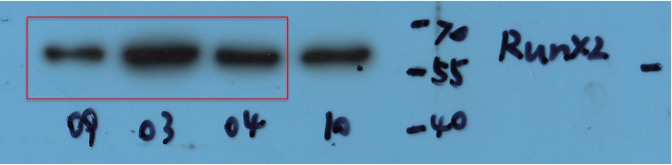


**VEGFA**


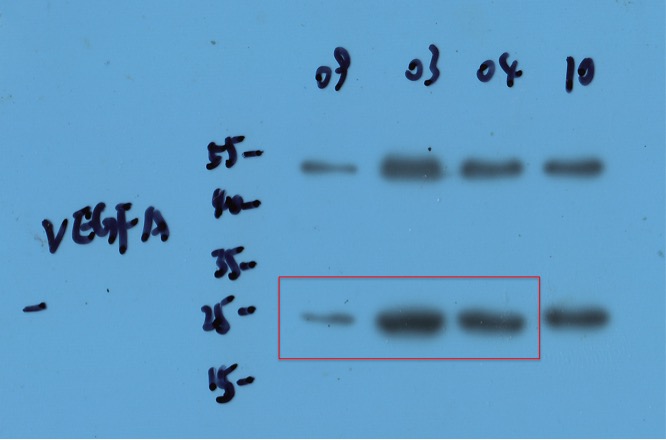


**TGF-β1**


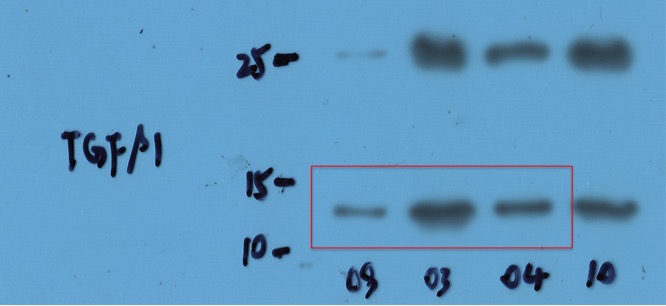


**actin**


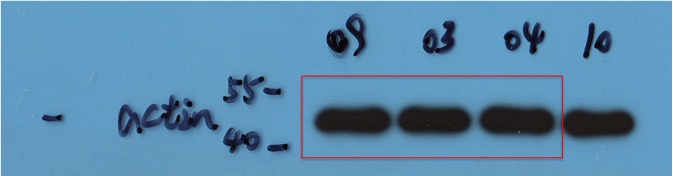


**09:** Control group; **03:** Fixation group; **04:** Non-fixation group; **10:** membrane around plates

**(2) 6 weeks postoperatively**

**ALP**

**
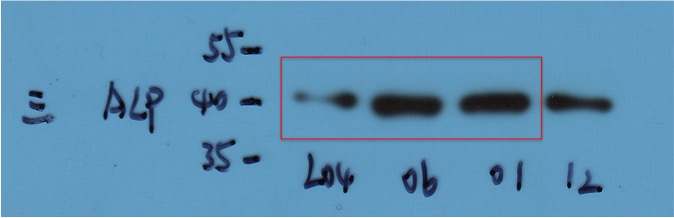
**

**RUNX2**

**
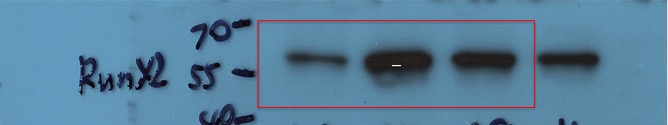
**

**VEGFA**


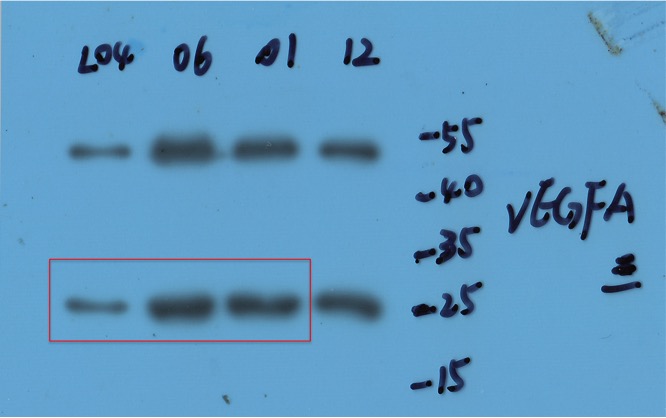


**TGF-β1**

**
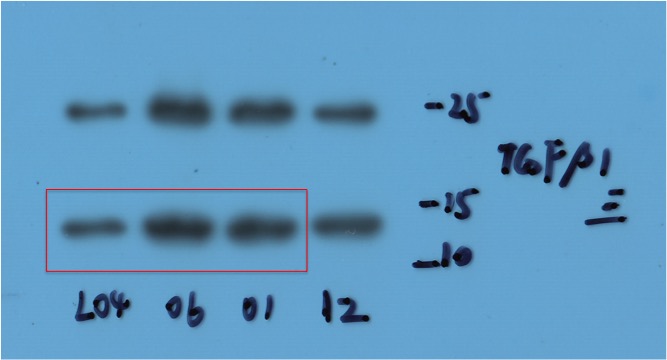
**

**actin**


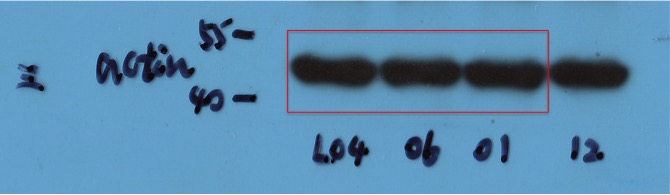


**L04:** Control group; **06:** Fixation group; **01:** Non-fixation group; **12:** membrane around plates
